# Functionalized Boron Carbide Nanoparticles as Active Boron Delivery Agents Dedicated to Boron Neutron Capture Therapy

**DOI:** 10.2147/IJN.S516534

**Published:** 2025-05-24

**Authors:** Anna Rudawska, Bożena Szermer-Olearnik, Agnieszka Szczygieł, Jagoda Mierzejewska, Katarzyna Węgierek-Ciura, Paulina Żeliszewska, Dawid Kozień, Monika Chaszczewska-Markowska, Zbigniew Adamczyk, Piotr Rusiniak, Katarzyna Wątor, Andrzej Rapak, Zbigniew Pędzich, Elżbieta Pajtasz-Piasecka

**Affiliations:** 1 Hirszfeld Institute of Immunology and Experimental Therapy, Polish Academy of Sciences, Wrocław, Poland; 2 Jerzy Haber Institute of Catalysis and Surface Chemistry, Polish Academy of Sciences, Krakow, Poland; 3 Depatment of Materials Science and Ceramics, AGH University of Krakow, Krakow, Poland; 4 Department of Geology, Geophysics and Environmental Protection, AGH University of Krakow, Krakow, Poland

**Keywords:** boron carbide, nanoparticles, targeted functionalization, anticancer therapy, BNCT

## Abstract

**Introduction:**

Boron neutron capture therapy (BNCT) is a promising targeted radiotherapy that enables the treatment of cancers at the cellular level. The crucial aspect of BNCT are boron carriers, which should selectively reach cancer cells by delivering high concentrations of boron. Therefore, we propose the use of boron carbide (B_4_C) nanoparticles functionalized with antibodies directed against receptors overexpressed in cancer cells, such as the low-density lipoprotein receptor (LDLR) and the epidermal growth factor receptor (EGFR).

**Methods:**

Hydrodynamic diameter measurements confirmed the stability of functionalized B_4_C nanoparticles in culture media during biological tests lasting up to 72 hours. The toxicity of the nanoparticles was assessed using the MTT assay and BrdU cell cycle assay on three types of cancer cells (PC-3, T98G, and SCC-25) with different levels of LDLR and EGFR surface expression. The uptake of functionalized B_4_C nanoparticles by cancer cells was assessed based on flow cytometry, fluorescence microscopy, and holotomography. Boron concentrations in cancer cells were quantified via ICP-MS.

**Results:**

Functionalized B_4_C nanoparticles showed even 2-fold higher interaction with SCC-25 cells characterized by the highest surface expression of both receptors than with PC-3 and T98G cells. Holotomographic imaging confirmed the greater intracellular uptake of functionalized B_4_C nanoparticles compared to unmodified B_4_C, providing further evidence for the selective targeting of boron to cancer cells. ICP-MS analyses showed that B_4_C anti-LDLR nanoparticles were the most effective in delivering a high boron concentration to cancer cells. Particularly in SCC-25 cells, the concentration was 9.58 ± 2.6 mg/L boron per million cells. The highest uptake by these cells was associated with a decrease in viability to 63% and a slight reduction in the percentage of cells in S phase after 24-hour exposure.

**Conclusion:**

Stable complexes of antibody-functionalized B_4_C nanoparticles were successfully obtained, demonstrating increased tropism towards cancer cells overexpressing LDLR and EGFR.

## Graphical Abstract

**Figure:**
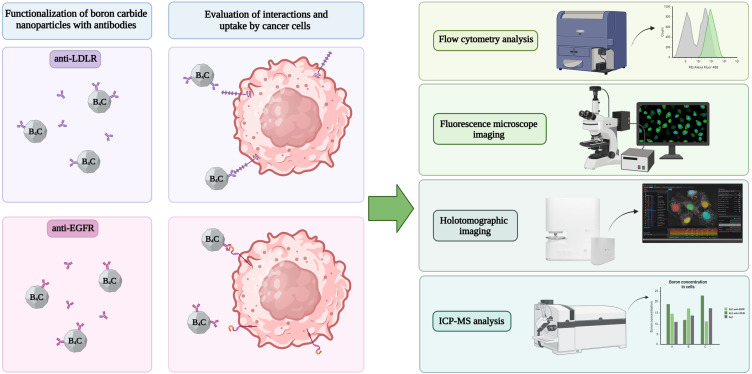

## Introduction

The most common type of radiation therapy uses an external beam with photons or other high-energy particles. The main disadvantage of this type of therapy is that radiation doses accumulate in healthy tissue before reaching the target. Therefore, the search for targeted therapies with reduced toxicity to healthy tissues that reach tumors located in hard-to-reach places is currently a major challenge in anticancer therapy. One promising strategy in this area is boron neutron capture therapy (BNCT), a tumor-selective form of radiotherapy. BNCT treatment involves administering the non-radioactive isotope boron-10 (^10^B) to patients and irradiating the tumor area with a neutron beam. Due to its large cross-section, ^10^B captures neutrons and generates alpha particles with high linear energy transfer (LET), causing damage to proteins, DNA, and RNA. The range of generated energy is limited to the size of a single cell (<10 μm), which allows for the destruction of cancer cells in which ^10^B has accumulated, while sparing normal tissues. Another advantage of BNCT over standard radiotherapy is using a neutron beam that is electrically neutral as it passes through the human body. Only when neutrons encounter the ^10^B isotope do electrical interactions occur, resulting in energy generation.

BNCT is used in patients with tumors that are resistant to conventional treatment, such as gliomas, melanomas, and head and neck cancers.^1^,^2^ Although the first clinical trial was conducted in 1951, BNCT is still not widely used in cancer treatment.^3^ The challenge is not only the limited availability of neutron sources, such as nuclear reactors and accelerators, but also the selectivity of compounds that will deliver a sufficient amount of ^10^B to cancer cells. To achieve the desired therapeutic effect in BNCT, boron compounds must meet certain requirements, such as low systemic toxicity, greater uptake by tumor than by normal tissues (ratio >3:1), delivery of >20 μg ^10^B per gram of tumor or >10^9^ atoms per cell, rapid removal from blood and normal tissues and retention in the tumor for the time required for neutron irradiation.^4^ Currently, two compounds are used in clinical trials: BPA ((L)-4-dihydroxy-borylphenylalanine) and BSH (di-sodium undecahydro-mercapto-closo-dodecacarborate). However, they have some limitations related to poor water solubility and bioavailability, which affect their distribution in the body. In addition, BPA and BSH show limited selectivity for different types of cancer, which reduces the effectiveness of the therapy. Due to the low content of boron atoms, they must be administered in large doses to provide the required therapeutic concentration of the ^10^B isotope, which may result in toxicity to normal tissues. Therefore, new boron compounds that will meet all requirements and ensure high effectiveness of BNCT are desired. Over the past few decades, many boron compounds have been designed and tested, including boronated amino acids, peptides, lipopeptides, boron clusters, boronated carbohydrates, nucleosides, porphyrins, polymers, polyamines, sugars, boron-containing immunoliposomes and liposomes, boronated antibodies, and boron nanoparticles.^2^,^4^,^5^

One promising boron agent is boron carbide (B_4_C), a boron-rich inorganic compound. The advantage of B_4_C is its large cross-section, making it an effective neutron absorber. Additionally, due to the use of multiple synthesis methods, B_4_C exhibits a wide range of stoichiometries, as well as polycrystalline and amorphous structures, characterized by a high boron content. This also enables the creation of boron carbide particles with the desired size and physicochemical properties. In previous studies, our team synthesized nanometric boron carbide and evaluated its biological activity, confirming its potential use in BNCT. Additionally, detailed physicochemical characterization enabled us to conclude that the surface of the obtained nanoparticles can be modified with functional groups, allowing for targeted functionalization.^6^,^7^ We also demonstrated significant uptake of boron carbide nanoparticles by murine macrophages, which served as cellular carriers of B_4_C for migrating and delivering boron to the tumor environment.^8^ In this work, we wanted to investigate another way of delivering boron carbide to cancer cells, namely by targeting receptors that are overexpressed in tumors. Therefore, nanometric boron carbide was functionalized using monoclonal antibodies against low-density lipoprotein receptor (LDLR) and epidermal growth factor receptor (EGFR). Antibiotics deserve special attention among the molecules used in targeted delivery systems due to their unique in vivo properties and high specificity.^9^ Moreover, this functionalization is original and innovative because such surface modifications of B_4_C have not been described yet. Our results provide evidence that functionalized boron carbide nanoparticles selectively deliver high concentrations of boron to LDLR- and EGFR-expressing cancer cells, making them promising boron carriers for BNCT.

The main methods of attaching antibodies to nanoparticles include physical and chemical adsorption. Physical adsorption enables the attachment of antibodies through various interactions, including electrostatic, hydrophobic, hydrogen, and ionic bonds, as well as van der Waals forces. This adsorption strategy is easy and convenient, but its effect is reversible, making it sensitive to environmental conditions and antibody displacement by ligands. Chemical adsorption relies on chemical conjugation to form stable covalent bonds between antibodies and nanoparticles. This method provides irreversible attachment of antibodies to nanoparticles but requires the use of chemicals that may affect the functionality of the antibodies and the properties of nanoparticles. Additionally, most covalent conjugations result in low efficiency and randomly oriented antibodies.^10^ In our study, anti-LDLR and anti-EGFR antibodies were immobilized on the surface of boron carbide by physical adsorption, based on electrostatic interactions resulting from the charge of the nanoparticles and the heterogeneous charge distribution in the antibody molecule.

LDLR is a transmembrane glycoprotein that is overexpressed in various types of cancers, including glioma, as well as liver, prostate, lung, breast, and colorectal cancers. The primary role of the LDLR is to regulate circulating low-density lipoprotein (LDL) levels and maintain cholesterol homeostasis. As cellular cholesterol levels decrease, the expression of LDLR increases, leading to the binding of circulating LDL by apolipoprotein B-100 (ApoB-100) or apolipoprotein E (ApoE) and their uptake through endocytosis. LDL is then transported to lysosomes, where it is hydrolyzed under the influence of lipases and low pH, resulting in the release of cholesterol and triglycerides. Due to hyperproliferation, cancer cells require more lipids, such as cholesterol, than normal cells. Therefore, the upregulation of LDLR enables rapid uptake of LDL through receptor-mediated endocytosis.^11^,^12^ The strategy for targeting LDLR most often includes attaching LDL or synthetic peptides containing the LDLR-binding site of the ApoB-100/ApoE ligand to drugs, carriers, or nanoparticles. Using anti-LDLR monoclonal antibodies to target cancer cells provides specific binding to extracellular domains responsible for interactions with LDL ligands. As a result, the inability to bind LDL may lead to increased levels of LDL.^13^ The efficacy of the LDLR-targeting strategy in anticancer therapy has been confirmed in the delivery of lipophilic derivations of doxorubicin, liposomes loaded with doxorubicin, and polybutylcyanoacrylate nanoparticles with doxorubicin or temozolomide to tumors. Additionally, the delivery of other cytostatics has been reported using LDLR-targeted chitosan and silica nanoparticles, as well as nanoparticles of gold, silver, zinc, platinum, or iron.^12^,^14^

The second receptor we selected for active delivery of B_4_C nanoparticles to cancer cells is EGFR. It is one of the most studied targets for anticancer therapy, mainly due to its overexpression in many types of cancers and correlation with poor prognosis. Clinically, the overexpression of this receptor occurs in 80–90% of head and neck cancers, 60% of glioblastoma multiforme, and 40% of prostate cancers.^15^ EGFR is a transmembrane protein belonging to the family of receptors with tyrosine kinase activity that exists as a monomer in the cell membrane. The receptor is activated upon ligand binding and then undergoes dimerization and autophosphorylation of its cytoplasmic domain, leading to the activation of the signaling pathway. Seven official EGFR ligands have been described, including epidermal growth factor (EGF), transforming growth factor-*α* (TGFA), heparin-binding EGF-like growth factor (HBEGF), betacellulin (BTC), amphiregulin (AREG), epiregulin (EPR), and epigen (EPGN). Under normal conditions, activated EGFR plays a crucial role in maintaining epithelial tissue, regulating cell proliferation, differentiation, division, migration, survival, and apoptosis.^16^ However, deregulation of the EGFR pathway results in cellular dysfunctions and pathologies, including increased proliferation, motility, angiogenesis, vascular mimicry, and invasiveness, which support tumor development.^17^ The delivery of boron by targeting EGFR has already been reported in BNCT. This concept involves the attachment of boron compounds to ligands such as EGF, antisense oligonucleotides against EGFR mRNA, and antibodies against EGFR. The use of anti-EGFR monoclonal antibodies to target cancer cells provides specific binding to the extracellular domain of EGFR, blocking ligand binding. As a result, receptor dimerization, autophosphorylation, and activation of signaling pathways may not occur.^18^ Anti-EGFR antibodies for targeted boron delivery to cancer cells have been described in combination with boron-containing dendrimers,^19^ Fc-binding peptide-dodecaborate,^20^ boron phosphate nanoparticles^21^, and others.

Active targeting of nanoparticles with antibodies against receptors overexpressed in tumors can effectively increase their delivery to cancer cells. Therefore, in the present study, we compared the ability of cancer cells with different levels of LDLR and EGFR expression to interact with and uptake boron carbide nanoparticles functionalized with anti-LDLR and anti-EGFR antibodies. Moreover, we provide evidence that B_4_C nanoparticles functionalized with antibodies, especially those targeting LDLR, are better engulfed and accumulated in cancer cells than unmodified B_4_C nanoparticles.

## Materials and Methods

### Characteristics of Boron Carbide Nanoparticles

The physicochemical characteristics of the boron carbide nanoparticles used in this work were previously determined using dynamic light scattering (DLS) and Laser Doppler Velocimetry (LDV) techniques, and the results were presented in the published paper by Kozień et al.^6^ Based on previous studies, we selected B_4_C nanoparticles of ~34 nm in size for functionalization, which were less toxic to cells than larger ones (~100 nm).^8^

### Functionalization of Boron Carbide Nanoparticles by Anti-LDLR and Anti-EGFR Antibodies Adsorption

Mouse anti-human LDLR (clone C7; Becton Dickinson) and mouse anti-human EGFR (clone AY13; BioLegend) antibodies were used to functionalize the boron carbide nanoparticles. Additionally, for flow cytometry and fluorescence microscopy studies, B_4_C nanoparticles were modified with fluorochrome-labeled antibodies: anti-LDLR PE (clone C7; Becton Dickinson) and anti-EGFR Alexa Fluor 488 (clone AY13; BioLegend).

The physical adsorption of anti-LDLR and anti-EGFR antibodies to B_4_C was determined by measuring the changes in electrophoretic mobility (zeta potential) caused by this process. The experiments were carried out according to the procedure previously applied to immunoglobulin G,^22^ which consists of three main steps:

- measurement of the electrophoretic mobility of bare boron carbide nanoparticles in a suspension with a concentration equal to 100 µg/mL (diluted from the suspension with a concentration of 1000 µg/mL),
- adsorption of anti-LDLR and anti-EGFR antibodies on B_4_C by mixing equal volumes of the suspensions, the adsorption time was 15 min,
- measurements of the electrophoretic mobility and diffusion coefficient of the anti-LDLR and anti-EGFR antibodies coated B_4_C nanoparticles.

### Calculations of the Average Number of Antibody Molecules per B_4_C Nanoparticle and the Probability Distribution

From the mass balance, the average number of antibody molecules per B_4_C nanoparticle can be calculated using the following formula:

(1) $$\bar N= {\pi \over 6}{{{N_{Av}}} \over {{M_w}}}{\rho _1}d_1^3\left({{{{c_a}} \over {{c_{B4C}}}}} \right)$$

where *N_Av_*= 6.02×10^23^ is the Avogadro number, *M_w_* is the molecular mass of the antibodies, ρ_1_= 2.5 [g/cm^3^] is the density of the B_4_C nanoparticle, *d*_1_ is the diameter of the B_4_C nanoparticle, *c_a_* is the initial concentration of the antibodies, and *c_B4C_* is the mass concentration of the B_4_C suspension.

One can calculate from Eq.(1) that for ${M_W} = 1.5 \times {10^5}g/mol$, *c_B4C_* = 1000 µg/mL and *d*_1_=34 nm one obtains the following linear dependence:

(2) $$\bar N= 2.08\,{c_a}$$

if *c_a_* is expressed in µg/mL or

(3) $$\bar N= 2.08 \times {10^{ - 6}}{M_w}{M_a}$$

if *c_a_* is expressed in nM.

Figure S1 shows the dependencies of $\bar N$ on calculated using Eq.(2) and Eq.(3). Additionally, Table S1 presents probability distributions of the number of antibody molecules per B_4_C nanoparticle calculated from the Poisson statistics for various values of $\bar N$.

### Stability of the Functionalized B_4_C Nanoparticles in Culture Media

Stability of the B_4_C nanoparticles functionalized with anti-LDLR and anti-EGFR antibodies was checked in three types of culture media: Ham’s F-12K supplemented with 10% FBS, DMEM/Ham’s F-12 supplemented with 10% FBS, and DMEM supplemented with 10% FBS.

Functionalized B_4_C nanoparticles at a concentration of 1000 µg/mL were mixed with each culture medium and incubated at 37°C for 7 days. During incubation, the hydrodynamic diameter was measured using the DLS method. Stability was determined for three independent samples, measured in five technical replicates.

### Cell Culture

SCC-25 cells from the human tongue squamous carcinoma line obtained from the American Type Culture Collection (ATCC; CRL-1628) were maintained in a 1:1 mixture of Dulbecco’s modified Eagle’s medium (DMEM; ATCC) and Ham’s F-12 medium (Gibco) containing 100 U/mL penicillin, 100 mg/mL streptomycin, 0.5 mM sodium pyruvate (all from Sigma-Aldrich), 15 mM HEPES (Gibco), 400 ng/mL hydrocortisone (Sigma-Aldrich) and 10% fetal bovine serum (FBS; Sigma-Aldrich).

PC-3 cells from the human prostate adenocarcinoma line derived from the European Collection of Authenticated Cell Cultures (ECACC; 90112714) were cultured in Ham’s F-12K medium (Gibco) supplemented with 100 U/mL penicillin, 100 mg/mL streptomycin, and 10% FBS (all from Sigma-Aldrich).

T98G cells from the human glioblastoma multiforme line obtained from ATCC (CRL-1690) were maintained in Eagle’s minimum essential medium (EMEM; ATCC) with the addition of 100 U/mL penicillin, 100 mg/mL streptomycin, and 10% FBS (all from Sigma-Aldrich).

All cell cultures were maintained in a NUAIRE CO_2_ incubator (37°C, 5% CO_2_, 95% humidity) at the Hirszfeld Institute of Immunology and Experimental Therapy, Polish Academy of Sciences, Wrocław, Poland.

### Assessment of the Expression Level of Receptors on the Cell Surface

SCC-25, PC-3, and T98G cells were centrifuged twice for 7 minutes at 192 × g and 4°C in phosphate-buffered saline (PBS). Next, the cells from each line were stained separately with an anti-LDLR PE antibody (clone C7; Becton Dickinson) and the appropriate IgG2b PE isotype control (clone 27–35; Becton Dickinson), as well as with an anti-EGFR APC antibody (clone AY13; BioLegend) and the IgG1 APC isotype control (clone MOPC-21; BioLegend) for 45 minutes at room temperature in the dark. The surface expression of LDLR and EGFR was analyzed using the LSRFortessa with Diva software (Becton Dickinson). Histograms of the flow cytometry analysis results were generated using NovoExpress software 1.3.0 (ACEA Biosciences, Inc). The assessment of receptor expression was conducted in two independent experiments, each in triplicate.

### MTT Viability Assay

SCC-25 (1 × 10^4^ cells/well), PC-3 (0.75 × 10^4^ cells/well) and T98G (0.5 × 10^4^ cells/well) cells were placed in 96-well plates. After 24 hours, unmodified B_4_C preparation and B_4_C functionalized with anti-LDLR and anti-EGFR antibodies (B_4_C anti-LDLR, B_4_C anti-EGFR) were added at concentrations ranging from 1 to 400 µg/mL and incubated for 24 and 72 hours. Next, MTT dye (3-(4,5-dimethylthiazol-2-yl)-2,5-diphenyltetrazolium bromide; Sigma-Aldrich) was added for 4 hours and lysed overnight in lysis buffer (N,N-dimethylmethanamide, sodium dodecyl sulfate, and water) at 37°C. The absorbance of the solubilized formazan crystals was measured at 570 nm using a Thermo Labsystems Multiskan RC microplate reader (Thermo Fisher Scientific Inc). with Genesis Lite 3.05 software (Thermo Fisher Scientific Inc). The MTT assay was performed in two independent experiments, each in triplicate.

### BrdU Cell Cycle Assay

SCC-25, PC-3 and T98G cells (1 × 10^5^ cells/well in 24-well plates) were incubated with unmodified B_4_C and functionalized with anti-LDLR or anti-EGFR (B_4_C, B_4_C anti-LDLR, B_4_C anti-EGFR) at concentrations of 50 and 100 µg/mL for 24 hours. After this time, 32 µM bromodeoxyuridine (BrdU; Becton Dickinson) solution was added for 1 hour, and the cells were subsequently collected and centrifuged for 10 min at 500 × g. The pellets were suspended in 70% ethanol and stored at −20°C. In the next step, the cell samples were prepared for staining with an anti-BrdU-FITC antibody (Becton Dickinson) according to the previously described protocol.^8^ The samples were analyzed using the LSRFortessa with Diva software (Becton Dickinson). The flow cytometry analysis scheme was performed using NovoExpress software version 1.3.0 (ACEA Biosciences, Inc). A BrdU cell cycle assay was conducted in two independent experiments, each in quadruplicate.

### Evaluation of Cell Interactions with Functionalized Nanoparticles by Flow Cytometry

SCC-25, PC-3, and T98G cells (1 × 10^5^ cells/well in 24-well plates) were incubated with B_4_C anti-LDLR labeled with PE and B_4_C anti-EGFR labeled with Alexa Fluor 488 at concentrations of 50 and 100 µg/mL for 4 and 24 hours. After this time, the cells were collected, centrifuged (7 min, 192 × g, 4°C), and suspended in PBS supplemented with 2 mM EDTA and 2.5% FBS (Sigma-Aldrich). DAPI dye (Thermo Fisher Scientific Inc). was added to each tube to eliminate dead cells. The mean fluorescence intensity (MFI) was analyzed using the LSRFortessa with Diva software (Becton Dickinson). Histograms were prepared in NovoExpress 1.3.0 software (ACEA Biosciences, Inc). The uptake of functionalized B_4_C nanoparticles was assessed in two independent experiments, each in triplicate.

### Fluorescence Microscopy

SCC-25, PC-3 and T98G cells (0.5 × 10^4^ cells/well) were placed in a black 96-well plate (Greiner Bio-One) and incubated with 100 µg/mL B_4_C anti-LDLR labeled with PE (B_4_C anti-LDLR-PE) and 100 µg/mL B_4_C anti-EGFR labeled with Alexa Fluor 488 (B_4_C anti-EGFR-AF488) for 24 hours. After exposure to B_4_C anti-LDLR-PE, live cell imaging was performed using an Axio Observer inverted microscope equipped with Zen Blue software (Zeiss).

After incubation with B_4_C anti-EGFR-AF488, the cells were fixed with 4% paraformaldehyde (Sigma-Aldrich) for 20 minutes, washed three times with PBS, and then permeabilized with a 0.25% Triton X-100 solution (Sigma-Aldrich) for 15 minutes. After this time, the cells were washed again with PBS and stained with DAPI dye (Thermo Fisher Scientific Inc). at a concentration of 0.1 µg/mL for 15 minutes. The cells were visualized using an Olympus IX81 inverted fluorescence microscope.

### Holotomography

SCC-25, PC-3, and T98G cells were seeded on a TomoDish (Altium) at a density of 1×10^5^ cells in 3 mL of culture medium. The next day, B_4_C, B_4_C anti-LDLR, and B_4_C anti-EGFR preparations at a concentration of 100 µg/mL were added for 24 hours. After this time, the culture medium containing noninternalized nanoparticles was discarded, and a fresh culture medium was added. The cells were visualized with an HT-2H commercial optical diffraction tomography microscope (Tomocube Inc). A 3D refractive index (RI) tomogram was created from multiple 2D holographic images acquired from 49 illumination conditions. The diffracted beams from the samples were collected using a high numerical aperture (NA = 1.2) objective lens UPLSAP 60XW (Olympus). The off-axis hologram was captured using a Blackfly S BFS-U3-28S5M complementary sCMOS image sensor (FLIR Systems Inc). The data were visualized and analyzed using TomoStudio HT-2H Gen3-3.3.9 software (Tomocube Inc). Dry cell mass was measured for an average of 10 cells per group.

### Analysis of the Boron Content in Cells Using ICP-MS

#### Microwave Digestion of Biological Material

The biological material under investigation had to be dissolved in a homogeneous solution before instrumental analyses. For this purpose, a modern ultraWAVE mineralization system (Milestone Srl) was applied. Before sample digestion, the material was well-mixed using a vortex mixer. Then, approximately 0.5 g of each material was sampled and transferred to Teflon vessels, into which 5 mL of concentrated 67% ultrapure nitric acid (V) (NORMATOM, Ultrapure for trace metal analysis) was added. In the next step, the Teflon vessels containing the samples were placed in a microwave mineralizer. The mineralization program included a 25-minute ramp-up to 250°C and a 20-minute hold at the target temperature. The microwave power was set to 1500 W. After digestion, the solutions were quantitatively transferred to 10 mL polypropylene vessels (avoiding borosilicate glassware, which could release boron into the solution), where they were diluted with deionized water obtained from the Direct-Q 3 UV system (Merck Millipore) to 10 g. Prepared samples were subjected to instrumental analysis for the determination of the total boron concentration in the biological material.

#### Instrumental Analysis of the Boron Content by ICP-MS

After microwave digestion, the biological material, both with and without the addition of boron carbide, was analyzed using the inductively coupled plasma mass spectrometry (ICP-MS) technique on the iCAP RQ (C2) instrument of Thermo Fisher Scientific in accordance with the ISO 17294–2 standard.^23^ During the analysis, a five-point calibration curve (including a blank sample) was established, covering concentrations ranging from 5 to 500 µg/L. Both the certified boron reference materials used for the calibration curve and the laboratory control samples used to control the calibration curve were produced in accordance with ISO 17034 standards.^24^ The certified reference materials used were Boron B - 10 g/L in H_2_O for ICP (CPAchem Ltd) and ICP multielement standard solution IV (Supelco Analytical Products). In the analysis, the ^10^B isotope was used for quantitative measurements, with a natural abundance in the environment of approximately 20%. Based on the determinations made in this study, the estimated limit of quantification was 1 µg/L, and the measurement uncertainty associated with boron determination by the ICP-MS technique was 16%. Subsequently, the results from the instrumental analyses were appropriately adjusted considering the mass of the sample taken for mineralization and the final mass after dilution. Measurements were performed on three independent biological samples for each cell type and preparation.

### Statistical Analysis

All data were analyzed using GraphPad Prism 8 software (GraphPad Software). The D’Agostino−Pearson omnibus test confirmed the normality of residuals. For dry cell mass measured by holotomography, the data were consistent with a Gaussian distribution and had equal standard deviation (SD) values. Therefore, statistical significance was calculated using the parametric one-way ANOVA followed by Tukey’s multiple comparison post-hoc test. Similarly, the results of the degree of nanoparticle interaction with cells determined by flow cytometry and boron concentration in cells data were consistent with a Gaussian distribution and had equal SD values, and the statistical significance was calculated using the two-way ANOVA followed by Tukey’s multiple comparisons post-hoc test. LDLR and EGFR surface expression data were consistent with a Gaussian distribution, but SD values were not equal, and the Brown−Forsythe and Welch ANOVA test, followed by Dunnett’s T3 multiple comparisons post-hoc test, was performed. The type of statistical analysis used is described in the captions under the figures. All statistically significant differences are presented in graphs when p<0.05; otherwise, the differences were not significant.

## Results

### Evaluation of Antibody Adsorption on B_4_C Nanoparticles

For surface modification, we selected a preparation in which the average size of the boron carbide nanoparticles was approximately 34 nm. In our previous study, this B_4_C preparation proved to be less toxic to cells than the preparation containing nanoparticles of about 100 nm.^6^ Additionally, smaller nanoparticles have a longer half-life in circulation and penetrate membranes more effectively, which is more promising for intravenous administration in future in vivo studies.^25^ Physical adsorption of antibody was carried out according to the procedure previously applied for immunoglobulin G.^22^ The changes in electrophoretic mobility (zeta potential) caused by this process allowed for the assessment of whether surface modification occurred and to what extent. Specifically, with increasing concentrations of antibodies added to the B_4_C suspension, increases in the zeta potential and hydrodynamic diameter were observed. This phenomenon was interpreted as the adsorption of antibodies on the boron carbide surface.

In the next step, the average number of antibody molecules adsorbed per B_4_C nanoparticle was calculated, and the probability distribution was assessed. The number of antibody molecules per boron carbide particle was calculated using the mass balance equation (Eq. (2)). As can be seen from analyzing Eq.(1), the number of adsorbed molecules depends on the molecular weight of the antibodies. Since the molar masses of mouse anti-human LDLR and mouse anti-human EGFR are similar (approximately 160 kDa), the calculations for both types of molecules are analogous. In these studies, antibody solutions with a concentration of 32 nM were used, which, according to theoretical calculations, indicates that statistically, one antibody molecule corresponds to one B_4_C particle (Table S1).

### Stability of Functionalized Boron Carbide Nanoparticles

**Figure 1:**
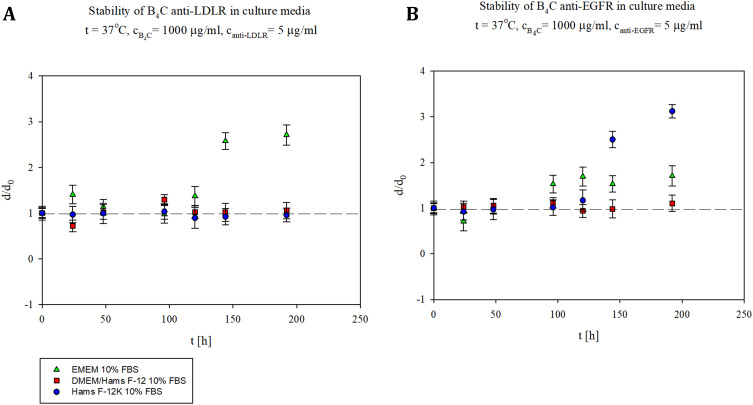
Stability of B_4_C nanoparticles functionalized with (**A**) anti-LDLR and (**B**) anti-EGFR antibodies expressed as the dependence of the normalized hydrodynamic diameter on time in various culture media. The points denote the experimental results obtained for the EMEM supplemented with 10% FBS (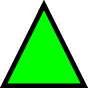), a 1:1 mixture of DMEM and Ham’s F-12 medium with 10% FBS (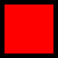), and Ham’s F-12K medium with 10% FBS (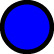). The B_4_C bulk concentration was 1000 µg/mL, and the anti-EGFR and anti-LDLR antibodies bulk concentration was 5 µg/mL. The results are expressed as the means ± SD calculated for three independent samples, measured in five technical replicates.

Stability of B_4_C nanoparticles modified with anti-LDLR and anti-EGFR antibodies was checked in three types of culture media used for further biological tests: Ham’s F-12K supplemented with 10% FBS, DMEM/Ham’s F-12 supplemented with 10% FBS, and DMEM supplemented with 10% FBS. The functionalized B_4_C nanoparticles at a concentration of 1000 µg/mL were mixed with three types of culture media and then incubated at 37°C for 7 days. As shown in Figure 1A, the B_4_C anti-LDLR nanoparticles remained stable for approximately 120 hours in all culture media. Above this time, we observed nanoparticle aggregation in EMEM supplemented with 10% FBS, while in the other two media, the complex remained stable. However, as shown in Figure 1B, the nanoparticles functionalized with anti-EGFR were stable for approximately 100 hours in all the culture media. Above 100 hours, the complex remained stable only in DMEM/Ham’s F-12 supplemented with 10% FBS, whereas aggregation of nanoparticles was observed in the other two media. The results confirmed the stability of the functionalized B_4_C nanoparticles during biological tests lasting up to 72 hours.

### Surface Expression Levels of LDLR and EGFR

**Figure 2:**
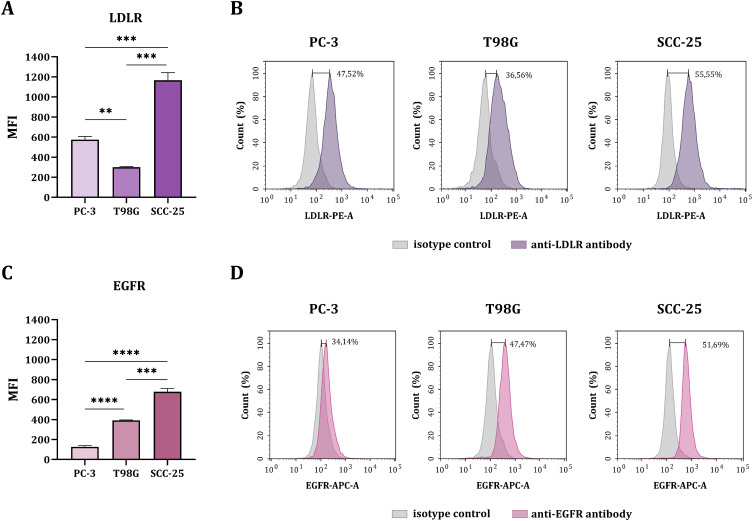
Expression of LDLR and EGFR on the surface of PC-3, T98G, and SCC-25 cancer cells. (**A**) Mean fluorescence intensity (MFI) of LDLR on the cancer cell surface. (**B**) Histograms of the fluorescence intensity of cancer cells stained with the isotype control and anti-LDLR-PE antibody. (**C**) MFI of EGFR on the cancer cell surface. (**D**) The histograms of fluorescence intensity of cancer cells stained with isotype control and anti-EGFR-APC antibody. The MFI in the graphs expresses the fluorescence intensity of the tested groups after deducting the MFI value of the isotype control (**A** and **C**). The results are expressed as the means + SD calculated for two independent experiments, each in triplicate. The differences between groups were calculated using the Brown‑Forsythe and Welch ANOVA, followed by Dunnett’s T3 multiple comparisons post‑hoc test (**p < 0.01; ***p < 0.001; ****p < 0.0001).

In the first stage of the biological studies, the surface expression of LDLR and EGFR on human cancer cells was assessed using flow cytometry. For this purpose, three cancer cell lines were selected: PC-3 prostate adenocarcinoma, T98G glioblastoma multiforme, and SCC-25 tongue carcinoma. The mean fluorescence intensity (MFI) shown in the graphs was calculated by subtracting the MFI of the appropriate isotype control. In the case of LDLR, the highest surface expression was demonstrated by SCC-25 cells, which was statistically significant in relation to the other cells tested (Figure 2A and B). T98G cells had the lowest LDLR expression. Regarding EGFR, SCC-25 cells exhibited the statistically significantly highest surface expression of this receptor, whereas PC-3 cells showed the lowest EGFR expression (Figure 2C and D). Thus, the highest expression of both receptors was observed in SCC-25 cells.

Based on this assay, we identified differences in the expression levels of the receptors on cancer cells from selected lines. We showed that these levels depended on the type of cancer. Nevertheless, the analysis of expression differences provided a good model for studying the degree of cell interaction with modified nanoparticles.

### Toxicity of the Boron Carbide Preparations

**Figure 3:**
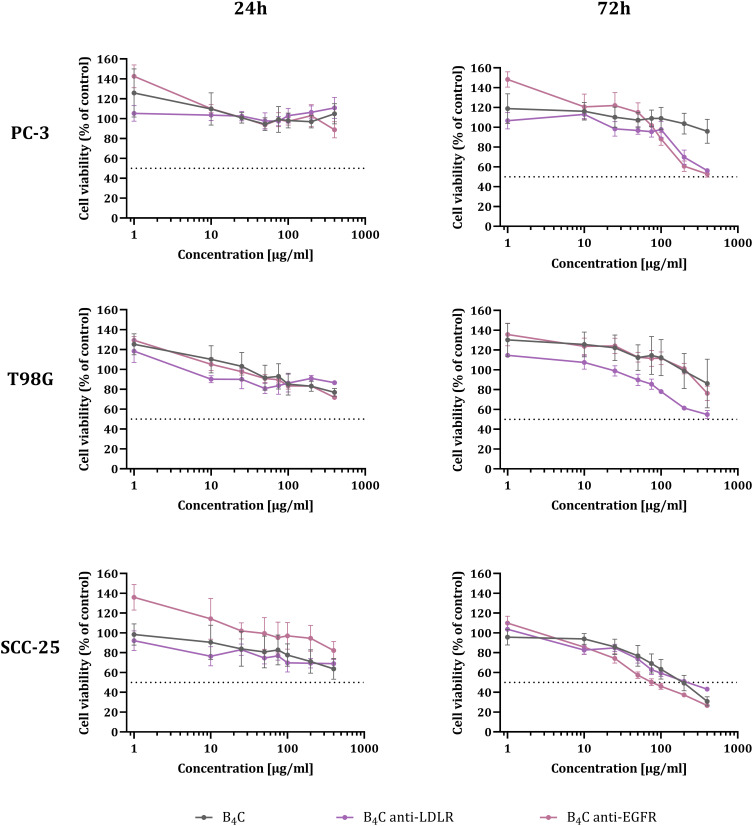
Effect of unmodified and functionalized boron carbide with anti-LDLR and anti-EGFR antibodies on the viability of human PC-3, T98G, and SCC-25 cancer cells after 24 and 72 hours of exposure. The graphs represent the percentage of viable cells relative to untreated control cells (Control = 100%). The results are expressed as the means ± SD calculated for two independent experiments, each in triplicate.

Boron carbide nanoparticles functionalized with antibodies were evaluated for their toxic effect on PC-3, T98G, and SCC-25 cancer cells. For this purpose, cell viability was assessed after 24 and 72 hours of exposure to B_4_C anti-LDLR, B_4_C anti-EGFR, and unmodified B_4_C as a control using the MTT assay. The toxicity of the tested boron carbide preparations increased depending on their concentration and incubation time with T98G and SCC-25 cells (Figure 3). The exception was PC-3 cells, whose viability after 24 hours of incubation with the B_4_C preparations was constant regardless of their concentration. Meanwhile, a toxicity assessment after 72 hours showed the differences. The viability of PC-3 and T98G cells remained above 50% in the tested concentration range from 1 to 400 µg/mL, indicating their low sensitivity to the B_4_C preparations. Whereas, SCC-25 cells, whose viability decreased to 26% after 72 hours of exposure to 400 µg/mL B_4_C nanoparticles, were the most sensitive to unmodified and functionalized B_4_C. In all the tested cells, B_4_C anti-LDLR and B_4_C anti-EGFR showed similar effects on cell viability as unmodified B_4_C, suggesting that the functionalization of B_4_C nanoparticles with antibodies does not affect their toxicity. Moreover, the results revealed that the higher the expression of the tested receptors on the cell surface, the stronger and faster the cytotoxic effect. The toxicity of the preparations proved to be low even after 72-hour exposure, which allowed choosing 24 hours for further studies of cell interactions with the analyzed nanoparticles.

### Effect of B_4_C Preparations on the Cell Cycle

**Figure 4:**
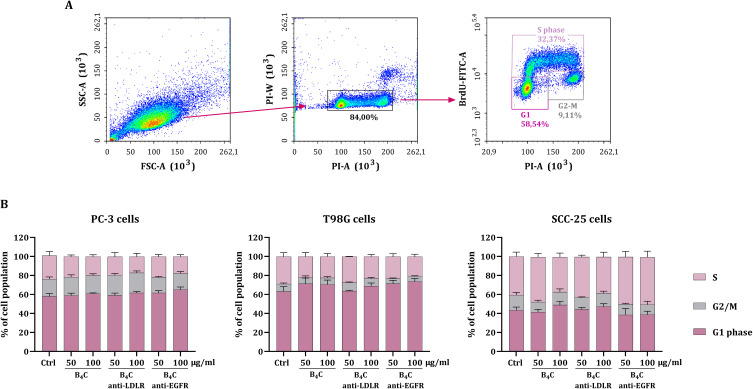
Effect of unmodified boron carbide nanoparticles and functionalized with anti-LDLR and anti-EGFR antibodies on the cell cycle phases of PC-3, T98G, and SCC-25 cells. (**A**) Scheme of flow cytometry analysis used to separate cell populations in the S, G2/M and G1 phases. (**B**) Percentages of PC-3, T98G, and SCC-25 cell populations in the S, G2/M and G1 phases after exposure to boron carbide preparations (B_4_C, B_4_C anti-LDLR, B_4_C anti-EGFR) at concentrations of 50 and 100 µg/mL compared to untreated control cells (Ctrl). The results are expressed as the means + SD calculated for two independent experiments, each in quadruplicate.

A BrdU assay was performed using flow cytometry to determine the relationship between the toxicity of the B_4_C preparations and changes in the cell cycle. For this purpose, PC-3, T98G, and SCC-25 cells were incubated for 24 hours with unmodified B_4_C and B_4_C modified with anti-LDLR and anti-EGFR antibodies at concentrations of 50 and 100 µg/mL. Figure 4A demonstrates a scheme of flow cytometry analysis to separate cell populations in the S, G2/M, and G1 phases. The changes in the cell cycle of all tested cancer cells after exposure to boron carbide preparations (B_4_C, B_4_C anti-LDLR, and B_4_C anti-EGFR) were minor (Figure 4B).

The smallest changes in cell cycle phases were visible in PC-3 cells, where the percentage of the cell population in cycle phases changed insignificantly. These results correlate with the observed lack of toxicity after 24 hours in the MTT assay. However, in T98G cells, all B_4_C preparations reduced the percentage of the cell population in the S phase while increasing the percentage of cells in the G1 phase compared to the untreated control. Additionally, for the B_4_C anti-LDLR preparation, this phenomenon was concentration-dependent. The greatest changes in cell cycle phases were observed for SCC-25 cells, where exposure to unmodified B_4_C and B_4_C anti-LDLR at a concentration of 100 µg/mL also resulted in a decrease in the percentage of cells in the S phase and an increase in the G1 phase. The opposite situation was observed after exposure of these cells to B_4_C anti-EGFR, which resulted in an increase in the percentage of the cell population in the S phase and a decrease in the G1 phase compared to the untreated control. Overall, the obtained results confirmed the low toxicity of the B_4_C preparations.

### Interactions and Uptake of Functionalized Boron Carbide Nanoparticles by Cancer Cells

#### Flow Cytometry Analysis

**Figure 5:**
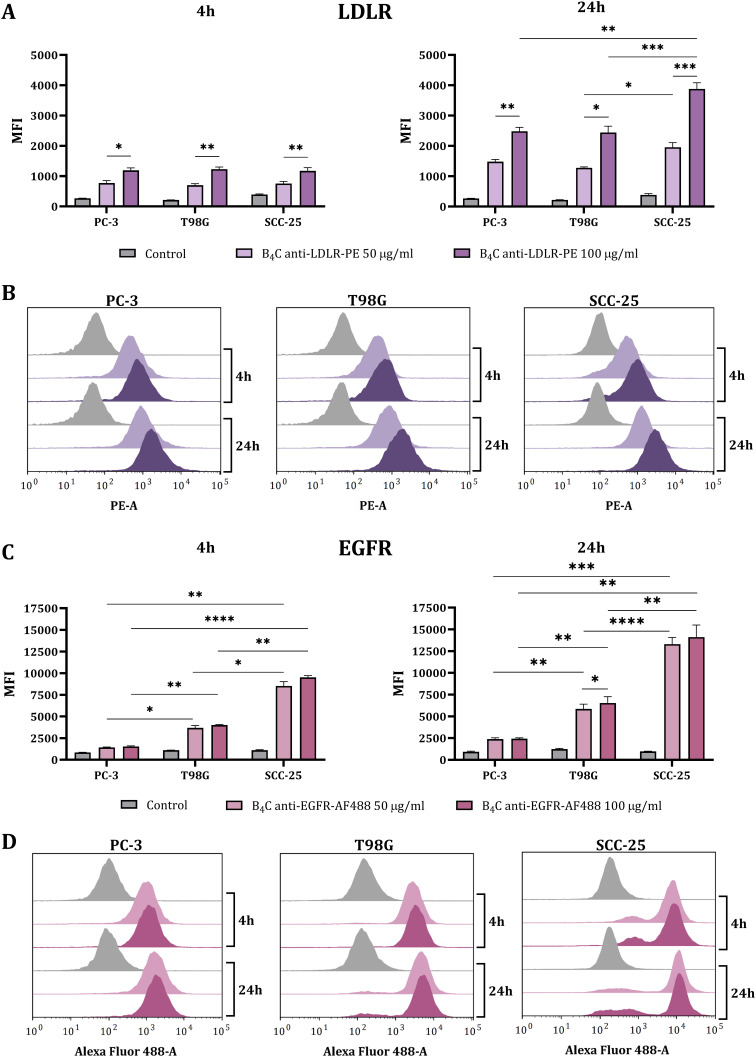
Interaction of PC-3, T98G, and SCC-25 cells with boron carbide preparations analyzed by flow cytometry. (**A**) Mean fluorescence intensity (MFI) of PC-3, T98G, and SCC-25 cells measured after 4 and 24 hours of exposure to B_4_C nanoparticles functionalized with PE-labeled anti-LDLR antibody (B_4_C anti-LDLR-PE) compared to untreated control cells. (**B**) Histograms of the fluorescence intensity of cancer cells after 4- and 24-hour incubations with B_4_C anti-LDLR-PE. (**C**) MFI of PC-3, T98G, and SCC-25 cells measured after 4 and 24 hours of exposure to B_4_C nanoparticles functionalized with Alexa Fluor 488-labeled anti-EGFR antibody (B_4_C anti-EGFR-AF488) compared to untreated control cells. (**D**) Histograms of the fluorescence intensity of cancer cells after 4- and 24-hour incubations with B_4_C anti-EGFR-AF488. The results are expressed as the means + SD calculated for two independent experiments, each in triplicate. The differences between groups were calculated using the two-way ANOVA followed by Tukey’s multiple comparison post-hoc test (*p < 0.05; **p < 0.01; ***p < 0.001; ****p < 0.0001).

After determining the toxicity effects of boron carbide preparations, the next stage of the research was to assess the interaction and uptake of the tested nanoparticles by the target cancer cells. First, an interaction evaluation of boron carbide nanoparticles labeled with fluorochromes was performed using flow cytometry. For this purpose, B_4_C nanoparticles functionalized with anti-LDLR antibodies and labeled with PE (B_4_C anti-LDLR-PE) and anti-EGFR antibodies labeled with Alexa Fluor 488 (B_4_C anti-EGFR-AF488) were incubated for 4 and 24 hours with PC-3, T98G, and SCC-25 cells. Analysis of the MFI after 4 hours of cell incubation with B_4_C-LDLR-PE revealed no statistically significant differences in the degree of interaction between the tested cells with different levels of LDLR surface expression (Figure 5A and B). Extending the incubation time to 24 hours resulted in differences in cell interactions with B_4_C LDLR-PE depending on the concentration and type of cells. SCC-25 cells exhibited the greatest ability to interact with B_4_C LDLR-PE, especially at a concentration of 100 µg/mL, compared to PC-3 and T98G cells, which was statistically significant. This observation correlated with these cells’ highest level of surface LDLR expression. In the case of B_4_C EGFR-AF488, statistically significant differences in MFI between cancer cell types were detected after 4 hours of incubation. However, this effect was not concentration-dependent (Figure 5C and D). A longer incubation time (24 hours) increased the MFI in all tested cells after exposure to B_4_C anti-EGFR-AF488, but the trends remained the same. SCC-25 cells showed the greatest interaction (highest MFI) with B_4_C anti-EGFR-AF488, whereas PC-3 cells presented the least interaction. The degree of cell interaction with the nanoparticles was correlated with the level of EGFR expression on their surface.

### Fluorescence Microscopy

**Figure 6:**
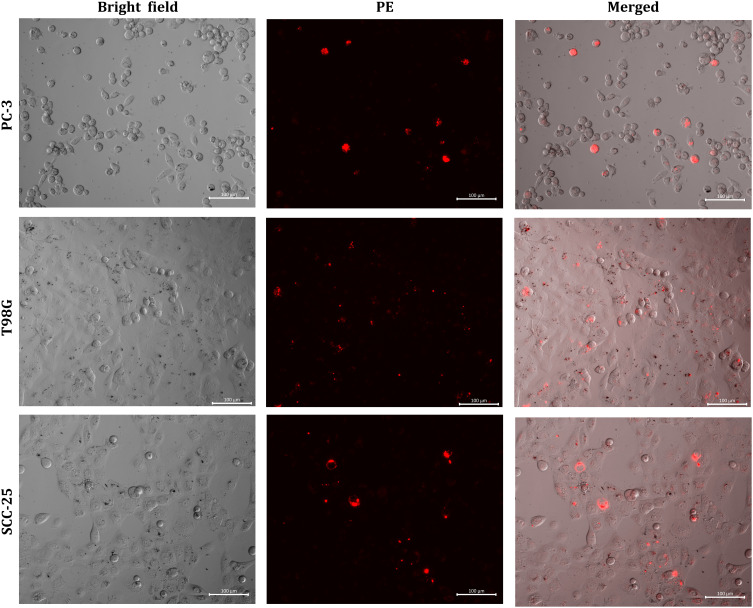
Fluorescence microscopy images showing the interaction of PC-3, T98G, and SCC-25 cells with B_4_C functionalized with PE-labeled anti-LDLR antibody after 24 hours of exposure. Live cell imaging was performed. The scale bar in all images is 100 µm.

The interactions and uptake of B_4_C anti-LDLR-PE and B_4_C anti-EGFR-AF488 nanoparticles by PC-3, T98G, and SCC-25 cells were also confirmed by fluorescence microscopy visualization. In the case of B_4_C anti-LDLR-PE, live cell imaging was performed (Figure 6). Bright field images showed that B_4_C anti-LDLR-PE nanoparticles accumulated inside the cells, which, in the fluorescence images, corresponds to the red glow coming from PE attached to B_4_C anti-LDLR.

**Figure 7:**
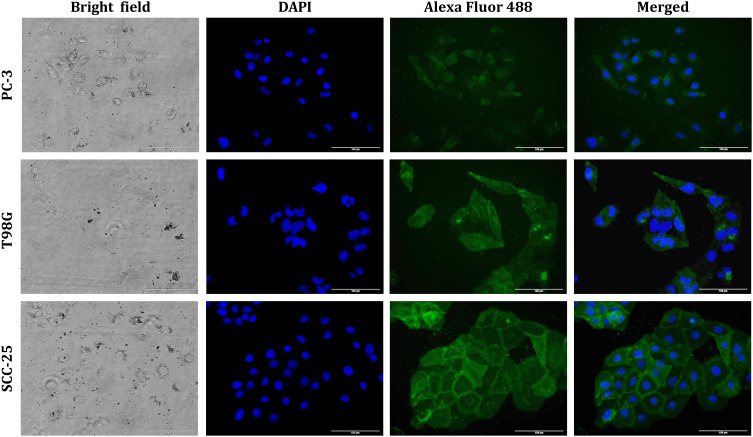
Fluorescence microscopy images showing the interaction of PC-3, T98G, and SCC-25 cells with B_4_C functionalized with Alexa Fluor 488-labeled anti-EGFR antibody after 24 hours of exposure. The cells were fixed, and the nuclei were stained with DAPI (blue fluorescence). The scale bar in all images is 100 µm.

Subsequently, cancer cells were fixed after incubation with B_4_C anti-EGFR-AF488 nanoparticles, and the nuclei were stained with DAPI. Accumulated nanoparticles were visualized inside cells in a bright field, and as the green fluorescence of Alexa Fluor 488-labeled B_4_C anti-EGFR (Figure 7). Similar to B_4_C anti-LDLR-PE nanoparticles, the highest fluorescence intensity was observed in SCC-25 cells, which was correlated with the highest level of EGFR expression on the surface of these cells. Thus, flow cytometry analysis and microscopic imaging confirmed the interaction of cells with functionalized B_4_C nanoparticles.

### Holotomography

**Figure 8:**
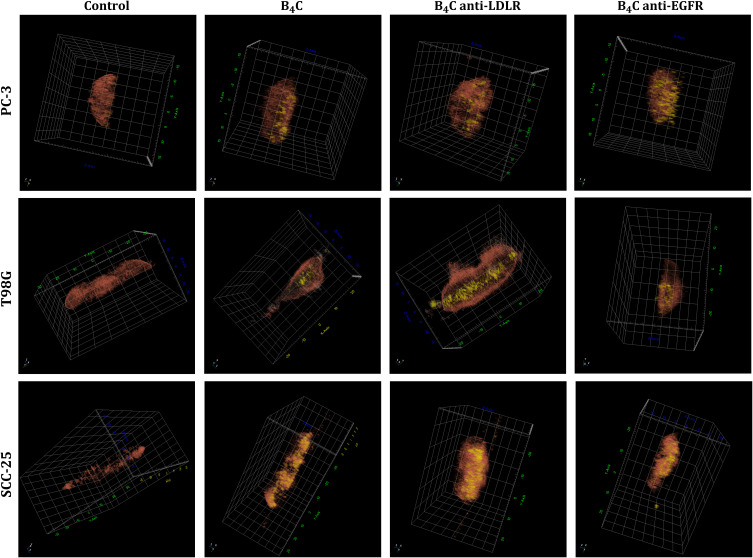
3D holotomographic visualization of PC-3, T98G, and SCC-25 cells with engulfed boron carbide nanoparticles (B_4_C, B_4_C anti-LDLR, B_4_C anti-EGFR) compared to untreated cells, using pseudo-coloring based on refractive index (RI) ranges. Brown structures have an RI of up to 1.38 and mark the interior of the cells (cytoplasm and organelles), while the yellow color corresponds to boron carbide nanoparticles with an RI above 1.38.

**Figure 9:**
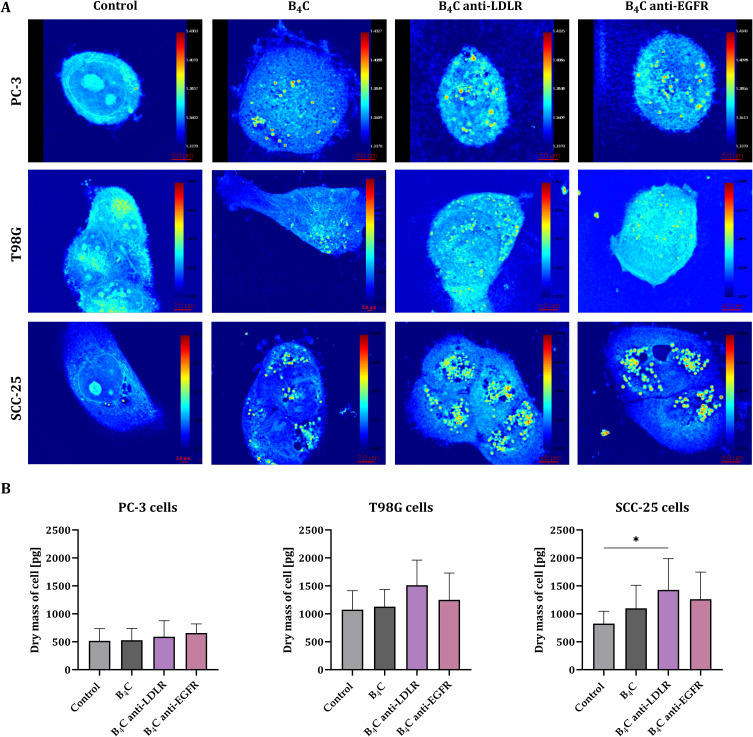
Holotomography. (**A**) Cross-sectional images of the refractive index distributions of PC-3, T98G, and SCC-25 cells after exposure to boron carbide nanoparticles (B_4_C, B_4_C anti-LDLR, and B_4_C anti-EGFR) compared to untreated cells. (**B**) The graphs show the dry mass of cells after incubation with boron carbide nanoparticles measured using a holotomography. For each preparation, an average of 10 individual cells was measured. The results are expressed as the means + SD of at least 7 measurements. The differences between groups were calculated using the one-way ANOVA followed by Tukey’s multiple comparison post-hoc test (*p < 0.05).

To confirm that the tested nanoparticles are not only able to bind to cell surface receptors but are also engulfed by cancer cells, we visualized the accumulation of B_4_C nanoparticles in PC-3, T98G, and SCC-25 cells using a holotomography. In addition, this device enabled the measurement of the refractive index (RI) of the tested cells. The results showed that the RI of cancer cells with unmodified and functionalized boron carbide nanoparticles was greater than that of untreated cells. This enabled the use of a pseudo-coloring technique that depends on differences in the refractive index between compartments of single cells. Figure 8 illustrates a 3D visualization of the structures present inside the cells. The organelles and cytoplasm are marked in brown (RI < 1.38), while the yellow color indicates boron carbide nanoparticles (RI > 1.38). Moreover, cross-sectional images of the RI distribution in cells after exposure to B_4_C nanoparticles are shown in Figure 9A. Holotomography images confirmed a significant accumulation of B_4_C, B_4_C anti-LDLR, and B_4_C anti-EGFR in all cancer cells compared to the untreated control cells. The most significant differences were observed for SCC-25 cells. Additionally, holotomography allowed for measurements of single-cell dry mass after exposure to each preparation (Figures 9B) and ten individual cells were measured to calculate the average. The greatest increase in dry mass after incubation with B_4_C nanoparticles was recorded in SCC-25 cells, especially after exposure to B_4_C anti-LDLR, which was statistically significant. Similarly, in T98G cells, the dry mass increased the most after incubation with B_4_C anti-LDLR compared to control cells. In contrast, the dry mass of PC-3 cells was almost unchanged after exposure to B_4_C nanoparticles.

**Figure 10:**
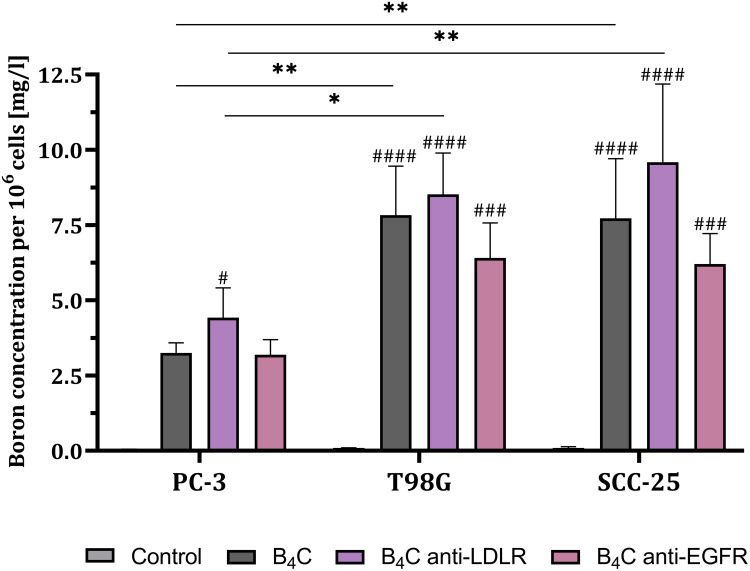
Boron concentration per 10^6^ PC-3, T98G, and SCC-25 cells after 24 hours of incubation with boron carbide preparations (B_4_C, B_4_C anti-LDLR, B_4_C anti-EGFR) compared to untreated control cells. The results are expressed as the means + SD calculated for three independent biological samples. The differences between groups were calculated using the two-way ANOVA followed by Tukey’s multiple comparison post-hoc test. The asterisks (*) presented in the graphs indicate statistically significant differences between the given groups; a hashtag (#) above a bar – between the given group and the untreated, control group (*/#p < 0.05; **p < 0.01; ###p < 0.001; ####p < 0.0001).

### Inductively Coupled Plasma Mass Spectrometry

In the final stage, the interaction and uptake of boron carbide nanoparticles by cancer cells were confirmed using a quantitative method. For this purpose, the boron concentration was determined in PC-3, T98G, and SCC-25 cells after 24 hours of exposure to B_4_C, B_4_C anti-LDLR, and B_4_C anti-EGFR nanoparticles using inductively coupled plasma mass spectrometry (ICP-MS). The results were calculated per 10^6^ cells (Figure 10). The analysis revealed statistically significant differences in boron concentrations in all tested cancer cells after incubation with B_4_C nanoparticles compared to untreated cells, where the boron content was negligible (<0.1 mg/L per 10^6^ cells). However, the boron concentration in all tested cells was the highest after exposure to B_4_C anti-LDLR and the lowest after incubation with B_4_C anti-EGFR. The highest boron concentration was detected in SCC-25 cells after incubation with B_4_C anti-LDLR nanoparticles (~9.58 mg/L per 10^6^ cells). The results confirmed that the highest surface expression of LDLR presented by these cells was accompanied by the greatest ability to interact with and accumulate functionalized boron carbide nanoparticles. Thus, the degree of cell loading with boron depends not only on the amount of preparation delivered to the tumor tissue but primarily on the rate of tumor cell proliferation and the high expression of specific molecules on the surface of these cells. Based on the obtained results, we postulate that functionalized B_4_C nanoparticles may be effective as a boron delivery agent for specific tumors.

## Discussion

One of the main challenges in cancer treatment is to reduce the harmful effects on normal tissues while effectively killing cancer cells. For many years, great emphasis has been placed on developing targeted anticancer therapies. In the case of radiotherapy, BNCT may be a good alternative as targeted therapy for cancers located in hard-to-reach places that do not respond to standard chemotherapy. The development of accelerator technologies that can generate a neutron beam of appropriate energy in clinical conditions will push BNCT forward. However, the BPA and BSH currently used in clinical trials do not meet all the requirements for BNCT carriers, which is a significant limitation. Therefore, the greatest challenge is to design compounds that highly selectively deliver boron to cancer cells without affecting normal tissues.

Our research focused on developing selective boron-rich nanoparticles for the targeted delivery of boron to cancer cells. For this purpose, we used physical adsorption to functionalize boron carbide nanoparticles with monoclonal antibodies against LDLR and EGFR, which are receptors commonly overexpressed in tumors. The next crucial step was to measure the hydrodynamic diameter of the functionalized B_4_C nanoparticles over time. The results showed no aggregation of nanoparticles up to 100 hours of incubation in different culture media. The aggregation of modified nanoparticles could affect their stability and bioavailability during biological tests. Additionally, this may result in limited boron delivery to the target cells.

To assess the toxicity of the functionalized B_4_C nanoparticles and their ability to interact with cells, we selected three cancer cell lines: PC-3, T98G, and SCC-25. Each cell line corresponds to a distinct type of tumor and is characterized by varying LDLR and EGFR expression levels. A cell proliferation assay confirmed that the functionalization of B_4_C nanoparticles with antibodies did not increase their toxicity toward PC-3, T98G, and SCC-25 cells compared to unmodified B_4_C. Moreover, the B_4_C preparations did not significantly inhibit cell proliferation after 24 hours. Toxicity was observed after 72 hours of incubation of cancer cells with nanoparticles at concentrations above 100 µg/mL. Despite this, SCC-25 cells proved to be the most sensitive to all the tested B_4_C preparations. The low toxicity of the tested preparations was also confirmed by cell cycle results, which showed slight changes in the cell cycle phases after 24 hours of exposure to B_4_C nanoparticles. Our previous research shows that boron carbide nanoparticles may exhibit different toxicities depending on the type and origin of the cells on which the tests are conducted.

The chemical composition and size of the nanoparticles are related to the synthesis method and determine their toxicity.^6^,^8^ This dependence was confirmed by other studies, including the work of Mortensen et al, which showed that boron carbide nanoparticles had no significant effect on the proliferation of B16 F10 melanoma cells. Only after neutron beam irradiation was 100% inhibition of cell proliferation observed.^26^ Similarly, Iwagami et al demonstrated that boron carbide encapsulated in a graphite layer was not toxic to human oral squamous cell carcinoma SAS cells after 24 and 48 hours of incubation.^27^ While in the work of Delong et al, boron carbide nanoparticles, already at a concentration of 25 µg/mL, showed toxicity to A375 melanoma cells after 24 hours of incubation.^28^

The functionalization of boron carbide nanoparticles with anti-LDLR and anti-EGFR antibodies is novel, as it has not been previously described. Studies to date have confirmed that boron carbide can be modified to target receptors overexpressed on cancer cells, enabling specific interaction and uptake. For example, in the work of Tsuji et al, boron carbide nanoparticles were coated with positively charged poly-L-lysine and negatively charged poly-γ-glutamic acid and additionally conjugated with transferrin to target the transferrin receptor. This study showed that these nanoparticles were extensively internalized by HeLa cervical carcinoma cells, which exhibit high surface expression of the transferrin receptor. In contrast, in normal rat kidney cells, modified B_4_C nanoparticles accumulated on the cell surface.^29^

However, the efficacy of delivering other boron compounds for BNCT by targeting LDLR and EGFR has been reported in several studies. For example, to target LDLR, Geninatti-Crich et al conjugated LDL with gadolinium/boron/ligand probes for more efficient delivery to HepG2 human liver cancer cells, B16 murine melanoma cells, and U87 human glioblastoma cells. In vitro studies showed the highest uptake by B16 cells, and in B16-tumor-bearing mice, high boron concentrations in tumors were detected as early as 4–6 hours after the administration of the compounds.^30^ Alberti et al prepared a boron/gadolinium (AT101) agent combined with LDL, which was taken up at the highest concentration by A549 human lung adenocarcinoma cells. Additionally, in mice with lung cancer and pulmonary metastases, slowing of tumor growth was observed after administration of this complex and neutron irradiation compared to controls.^31^ The same AT101-LDL complex also showed efficacy in delivering a therapeutic dose of boron to ZL34 mesothelioma cells, and following neutron irradiation, it reduced the tumor mass by approximately 80–85%.^32^

Studies also confirm the potential of using anti-EGFR antibodies to functionalize boron compounds dedicated to BNCT. For example, Wu et al demonstrated that a conjugate of boron-containing dendrimers and the anti-EGFR monoclonal antibody cetuximab was effectively delivered to tumors in F98 glioma-bearing rats.^19^ Similarly, Nakase et al attached Fc-binding peptide-dodecaborate to cetuximab and reported that the conjugate was recognized and accumulated on the plasma membrane via EGFR in F98G glioma cells.^20^ Additionally, Kuthala et al showed that boron phosphate nanoparticles modified with anti-EGFR antibody were taken up ∼5.8-fold more than nanoparticles without antibody functionalization by FaDu head and neck cancer cells.^21^

The most crucial step in our studies was to evaluate the degree of interaction and uptake of functionalized B_4_C nanoparticles by cancer cells and the effectiveness of supplying them with boron. Flow cytometry confirmed the interactions of functionalized B_4_C nanoparticles with cancer cells, which correlated with LDLR and EGFR expression levels on the cell surface. Additionally, fluorescence microscopy and holotomography enabled imaging of engulfed and accumulated modified nanoparticles inside the cells. However, the most important aspect was the assessment of the boron concentration within cancer cells using the ICP-MS method. This analysis confirmed that the highest boron concentration of 9.58 ± 2.6 mg/L per 10^6^ cells was present in SCC-25 cells after 24 hours of exposure to the B_4_C anti-LDLR. We assume that the highest uptake of the B_4_C anti-LDLR nanoparticles by SCC-25 cells is related to the highest surface expression of LDLR in these cells compared to other cancer cells. Moreover, the high uptake of B_4_C nanoparticles by SCC-25 cells also explains the observed toxicity toward these cells. Therefore, we can speculate that the transport of functionalized boron nanoparticles into the cell may occur via an active pathway through interaction with receptors on the cancer cell surface.

Based on the ICP-MS results, the B_4_C anti-EGFR nanoparticles were the least effective in delivering boron to cancer cells. However, with this method, we assessed the boron concentration only inside the cells, whereas a significant portion of the nanoparticles could be attached to the cell surface without entering the interior. One reason for this phenomenon may be the limitation related to the number of nanoparticles that can be engulfed by cells.^8^ Furthermore, the poor uptake efficiency of B_4_C anti-EGFR nanoparticles may also be due to the low antibody-to-nanoparticle ratio (1:1). Perhaps more effective functionalization would enable better interactions with cancer cells. Although functionalization of B_4_C nanoparticles with anti-EGFR antibodies was not as effective as in the case of anti-LDLR, the results provide evidence that boron carbide, as a boron-rich compound, can deliver boron at very high concentrations to cancer cells. Therefore, we believe that delivering the required boron concentration to tumor tissue to achieve a therapeutic effect after neutron beam irradiation will not be a limitation in future studies.

The observed uptake of unmodified B_4_C nanoparticles by cancer cells is a non-specific phenomenon. Surface modification aims to enhance the selectivity of these nanoparticles towards cancer cells, thereby reducing systemic toxicity and side effects. Moreover, we suppose that even if functionalized B_4_C nanoparticles do not enter cancer cells, but reach the tumor microenvironment and attach to the cell surface in large quantities, they may be sufficient for the success of BNCT. The boron concentrations obtained in our studies after incubation of cancer cells with functionalized B_4_C nanoparticles are higher than those provided by BPA and BSH. For example, in rat glioma F98 cells, after 24 hours of incubation with BPA, a maximum boron concentration of approximately 30 µg per 10^9^ cells was observed.^33^ Whereas after incubation of U-87 MG glioblastoma cells, FaDu pharynx cancer cells, and SAS tongue cancer cells with BPA at a concentration of 2000 μg/mL, the boron concentration was 11.1 ± 0.8, 23.6 ± 1.7, and 36.6 ± 1.2 ng/10^5^ cells, respectively.^34^ In contrast, BSH delivered 33.88 ng boron/10^6^ cells to A549 lung cancer cells.^35^

The obtained results provide a solid basis for further in vivo studies, but selecting the optimal dose of functionalized B_4_C nanoparticles is essential. Moreover, our studies may have clinical applications due to the correlation between LDLR and EGFR expression in immortalized cell lines and the expression of these receptors in tumors occurring in patients. Clinical data confirm the high expression of EGFR compared to normal tissues, especially in glioblastoma multiforme, as well as head and neck cancers, which include tongue squamous cell carcinoma, and slightly lower in prostate cancer. The same is true for LDLR, the expression of which is highest in head and neck cancers, slightly lower in gliomas, and lowest in prostate cancers compared to healthy tissues.^15^

## Conclusion

Our results confirmed that the degree of cell interaction with the B_4_C anti-LDLR and B_4_C anti-EGFR nanoparticles was correlated with the level of LDLR and EGFR expression on the surface of cancer cells. SCC-25 cells with the highest expression of both receptors showed the most significant ability to uptake functionalized nanoparticles. B_4_C anti-LDLR proved to be the most effective in delivering high concentrations of boron to cancer cells. The highest boron concentration was achieved in SCC-25 cells and was 9.58 ± 2.6 mg/L of boron per 10^6^ cells. The results provide a solid basis for further targeted modifications of nanoparticles dedicated to anticancer therapy.

## Data Availability

All generated and analyzed data have been incorporated into the manuscript.

## Abbreviations

ApoB-100, apolipoprotein B-100; ApoE, apolipoprotein E; AREG, amphiregulin; B_4_C, boron carbide; ^10^B, boron-10 isotope; BNCT, boron neutron capture therapy; BPA, (L)-4-dihydroxy-borylphenylalanine; BrdU, bromodeoxyuridine; BSH, di-sodium undecahydro-mercapto-closo-dodecacarborate; BTC, betacellulin; DLS, dynamic light scattering; EGF, epidermal growth factor; EGFR, epidermal growth factor receptor; EPGN, epigen; EPR, epiregulin; HBEGF, heparin-binding EGF-like growth factor; ICP-MS, inductively coupled plasma mass spectrometry; LDL, low-density lipoprotein; LDLR; low-density lipoprotein receptor; LDV, Laser Doppler Velocimetry; LET, high linear energy transfer; MFI, mean fluorescence intensity; TGFA, transforming growth factor-*α.*
